# Acute mental stress-induced alpha or beta-adrenergic reactivity patterns linked to unique cardiometabolic risk profiles

**DOI:** 10.1038/s41598-025-92961-2

**Published:** 2025-03-13

**Authors:** Annemarie Wentzel, Dewald Naudé, Roland von Känel

**Affiliations:** 1 Hypertension in Africa Research Team (HART), North-West University, Private Bag X6001, Potchefstroom, 2520 South Africa; 2 South African Medical Research Council Unit for Hypertension and Cardiovascular Disease, North-West University, Potchefstroom, South Africa; 3 Department of Consultation-Liaison Psychiatry and Psychosomatic Medicine, University Hospital, Zurich, Switzerland

**Keywords:** Acute mental stress, α-adrenergic response, β-adrenergic response, Cardiometabolic risk, Cardiovascular biology, Neurophysiology, Epidemiology, Outcomes research

## Abstract

Cardiometabolic risk may differ based on a stress-induced alpha(α)-adrenergic response versus a predominant beta(β)-adrenergic response. Whether these responses might serve as significant markers of distinct cardiometabolic risk profiles based on hemodynamic reactivity remain unknown. We (1) characterized predominant α-and β-adrenergic hemodynamic response patterns to acute mental stress; and (2) determined the cardiometabolic risk profile within predominant α-or β-adrenergic responders, irrespective of age, sex, or ethnicity. We included 117 South African teachers (aged 20–65 years) and administered an acute mental stress task (Color-Word-Conflict test) for one-minute. Participants’ hemodynamic response profiles were characterized as predominant α-adrenergic (decreases in cardiac output (CO) and Windkessel arterial compliance (C_wk_) (lowest quartile)) (*n* = 48) and β-adrenergic (increases in CO, C_wk_ (highest quartile)) responses (*n* = 69) via Finometer beat-to-beat hemodynamic monitoring. Ambulatory-BP was measured and the number of 24 H-ischemic events determined by ECG. Cardiometabolic markers were analyzed using fasting blood samples, and abnormal glucose tolerance (Abnl-GT), combining prediabetes and diabetes, was defined as glycated hemoglobin (HbA1c) ≥ 5.7% and/or fasting glucose > 100 mg/dL and/or diabetes medication usage. Predominant α-adrenergic responders presented with an overall poorer cardiometabolic profile, with higher levels of HbA1c, insulin, greater insulin resistance and higher total cholesterol and lower HDL-cholesterol. Adjusted analyses indicated that a predominant α-adrenergic profile had higher odds of central obesity (*P* = 0.031), low HDL-cholesterol (*P* = 0.042), 24-H-hypertension (*P* < 0.001), cardiac stress (*P* = 0.025), ischemic events (*P* = 0.048) and medium-to-high 10-year stroke probability (*P* < 0.001), compared to β-adrenergic responders. In the β-adrenergic responders, higher odds for ischemic events, stroke probability and Abnl-GT were found (all *P* ≤ 0.022), compared to α-adrenergic responders. Independent of age, sex or ethnicity, the risk profile identified in predominant α-adrenergic responders mainly involved the effects of a high-pressure system, cardiac stress, and ischemia. Whereas in predominant β-adrenergic responders, the risk profile pointed to a more metabolic and hyperperfusion injury-related cardiometabolic risk.

## Introduction

The hemodynamic result of stress and stress reactivity involves the activation of the sympathetic nervous system (SNS), leading to distinct hemodynamic adaptations and responses^1,2^. Sympathetic nervous system hyperactivity has been linked to an increase in cardiometabolic risk, including hypertension, cardiac structural remodeling, coronary artery disease (CAD), abnormal glucose tolerance (Abnl-GT), dyslipidemia and stroke^3–7^. Sustained, long-term SNS activation may also affect our ability to adequately respond to acute stressors, both mental and physical, resulting in a specific SNS-prompted hemodynamic reactivity pattern^7–9^. These effects are mediated via epinephrine or norepinephrine binding to alpha (α)- and/or beta (β)-adrenergic receptors. Although invasive microneurography can directly measure SNS activation in the skeletal muscle^10^, it cannot distinguish between α-or β-adrenergic receptor activation. However, one way to possibly assess such activation non-invasively, is by defining cardiovascular hemodynamic reactivity patterns, during acute stress application, that are the result of predominant α-adrenergic and/or β-adrenergic activation.

A mental stressor, such as the Stroop Color-Word-Conflict (STROOP-CWC) test, evokes a distinct SNS-induced hemodynamic response pattern^7,8,11,12^. Two predominant hemodynamic response profiles are the result of either a predominant α- or β-adrenergic receptor activation. A peripheral, vascular α-adrenergic response profile typically involves increases in diastolic blood pressure (DBP) and total peripheral resistance (TPR) and decreases in stroke volume (SV), cardiac output (CO) and arterial compliance (measured as Windkessel compliance (C_wk_)), due to α_1_-adrenergic-receptor activation in the peripheral vasculature^7^. In contrast, a central, cardiac β-adrenergic response is characterized by increases in systolic blood pressure (SBP), heart rate (HR), SV, CO, and decreases in TPR, due to β_1_-adrenergic (in the central cardiac tissue) and β_2_-adrenergic (in the blood vessels) receptor activation^7,8^. Activation of both α-and β adrenergic receptors are usually involved in an acute hemodynamic response, yet a predisposition for α-or β adrenergic activation may lead to altered hemodynamic reactivity, changing a normal physiological process into a response leading to increased cardiovascular and cardiometabolic risk.

A shift in autonomic activity, favoring SNS activity, is more often reported in individuals that show hallmarks of an α-adrenergic response, particularly increased TPR and decreased C_wk_^8^. These individuals’ hemodynamic characteristics of an α-adrenergic response are linked to an increased risk of CAD, cardiac stress, hypertension and ischemia, specifically in a Black South African population^7,8,13,14^. However, data on cardiometabolic risks linked to the hemodynamic pattern of β-adrenergic responders, which might also be linked to greater SNS activity, is limited, especially independently of age, sex or ethnicity. A plausible mechanism for risk in β-adrenergic responders may relate to hyperperfusion injury^15^, possibly contributing to ischemic heart injury and cardiac hypertrophy if β-adrenergic receptor activation is sustained for long periods of time^15,16^. A unique cardiometabolic risk profile may be linked to a predominant α-or β-adrenergic response, more so than a mixed adrenergic response. This may allude to pathophysiological pathways that link mental stress and specific hemodynamic reactivity during acute stress to cardiometabolic risk factors. One can postulate that as these hemodynamic response patterns relate to adrenergic receptor activation, the default pattern might be genetically predetermined, or subject to change based on sustained stress-exposure and/or effective stress control and adaptation. Indeed, excessive exposure to catecholamines, as occurs during SNS hyperactivity, may shift the receptor sensitivity in favor of an α_1_-receptor mediated response, therefore a more peripheral, vascular effect, rather than a central cardiac pattern, as observed in β_1_-adrenergic receptor activation^17,18^. Yet, the assumption can be made that regardless of the receptor and thus adrenergic-hemodynamic response predominance, a state of SNS imbalance may perpetuate increased cardiometabolic risk. No study has investigated the possibility that the predominance of an adrenergic *type* of hemodynamic response, hence predominantly α-or β-adrenergic, activated during acute stress, may be a unique cardiometabolic risk factor. Such novel knowledge might inform specific intervention strategies and unique risk stratification.

To our knowledge, this is the first study to characterize a predominant α-or β-adrenergic-hemodynamic response using non-invasive acute stress-induced hemodynamic measurements, and the describe the cardiometabolic risk profile associated with these specific hemodynamic responses while controlling for age, sex, and ethnicity. Therefore, we aimed to (1) characterize predominant α- or β-adrenergic response patterns to acute mental stress; and (2) describe the cardiometabolic and ischemic risk profile within predominant α-or β-adrenergic responders.

## Materials and methods

### Study design and participants

**Fig. 1:**
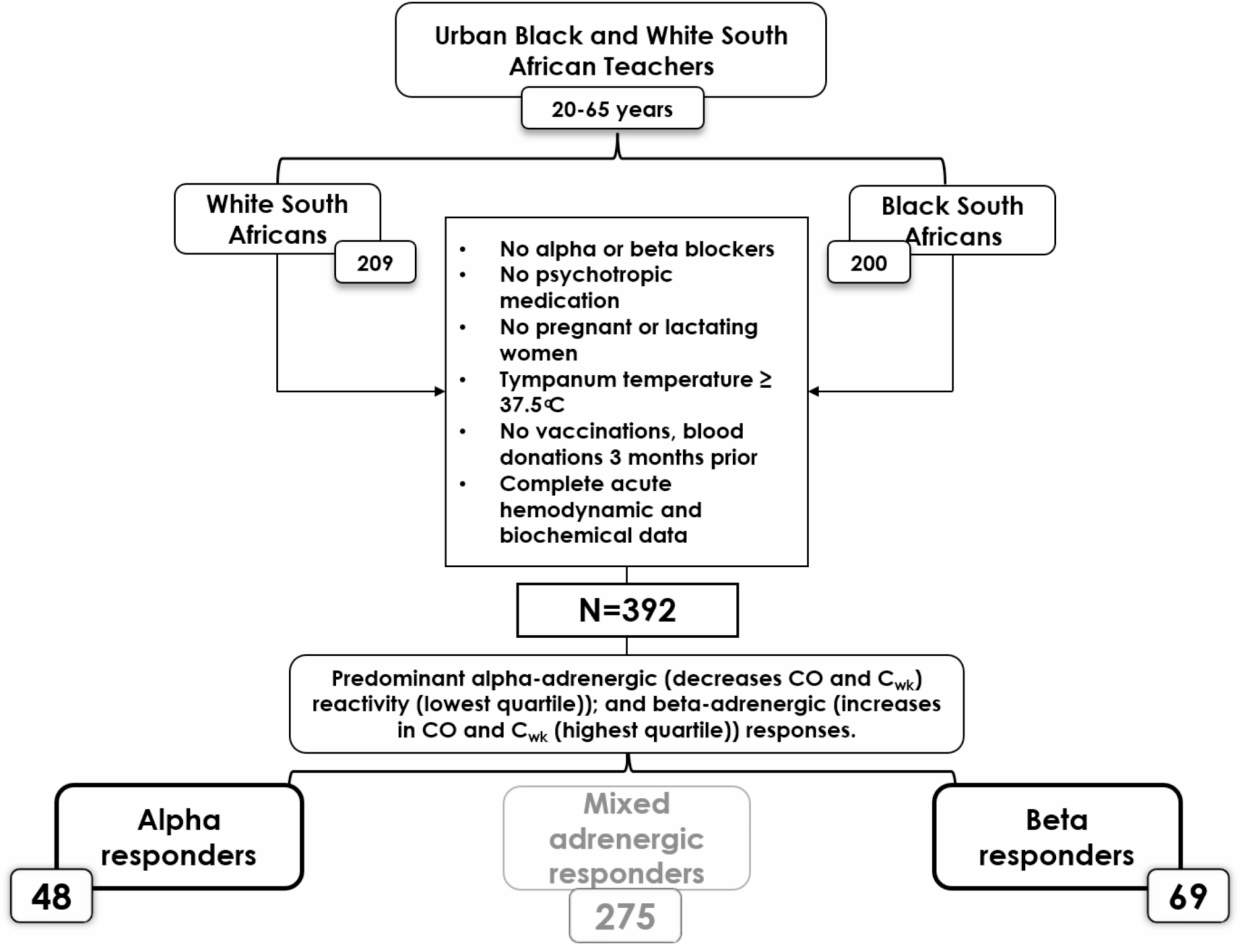
SABPA hemodynamic reactivity categorization. *∆%CO* cardiac output reactivity, *∆%Cwk* Windkessel arterial compliance reactivity.

The participants were recruited as part of phase 1 of the Sympathetic activity and Ambulatory Blood Pressure in Africans (SABPA) study^19^. Acute mental stress testing was only done in phase 1 of SABPA, and thus only the baseline phase was included in this study. The larger study sample comprised of urban male and female Black and White South African teachers (*N* = 409) from the North-West province of South Africa, aged between 20 and 65 years. The motivation behind the selection of schoolteachers was to obtain a socio-economic equated sample from a similar working environment, similar access to healthcare and work stress, however cultural differences could not be excluded or adjusted for. Originally, for SABPA a power analysis was conducted and determined (from previous studies) that a sample size of 50 to 416 would be sufficient to explain biological differences with a statistical power of 0.8 and level of significance of 0.05^19^. The exclusion criteria of the larger SABPA-study were any alpha/beta blocker users; pregnant or lactating women; tympanum temperatures greater than 37.5 °C; psychotropic medication usage and any blood donations 3 months prior to study inclusion. Furthermore, from this baseline, exploratory cohort, only those that had complete hemodynamic and cardiometabolic data and were novelly categorized as predominantly α-or β-adrenergic hemodynamic responders, to assess the unique cardiometabolic risk within these two adrenergic extremes, were included in this study (*N* = 117) (Fig. 1).

### Ethical considerations

The SABPA study obtained ethical approval from the Health Research Ethics Committee (HREC) of the North-West University (NWU) (NWU-00036-07-A6). Written informed consent was obtained from all volunteers prior to participation. All procedures and objectives were explained to the participants prior to their recruitment, and adhered to the applicable institutional guidelines and terms, as stated by the Declaration of Helsinki (2004), and this affiliated study also adhered to the updated Department of Health (DoH) South Africa’s guidelines on Ethics in Health Research in human participants (2024).

### General procedure of investigation

Clinical assessments were conducted over a two-day period during the baseline phase of SABPA^19^. Before 08h00 of the first clinical assessment day, participants were each fitted with an ambulatory blood pressure (ABPM) and 2-lead ECG monitor device (Cardiotens CE120^®^; Meditech CE120; Meditech, Budapest, Hungary). The device was programmed to record blood pressure (BP) measurements every 30 min during the day and every hour during the night. Participants subsequently continued with their normal daily activities, reporting any peculiarities such as nausea, headaches, visual disturbances, palpitations, fainting, stress, and physical activity, on the issued 24-hour diary cards. At 15h00, participants were transported to the NWU Metabolic Unit Research Facility for clinical measurements and demographic questionnaires, during which age, ethnicity, sex, medical history and medication usage were reported.

### Anthropometric and physical activity measurements

In a private, temperature-controlled room, two trained level II anthropometrists followed standard procedures to obtain body height, weight, and waist circumference (WC). All anthropometric measurements were performed according to guidelines as described by the International Society for the Advancement of Kinanthropometry (ISAK)^20^. Body surface area (BSA) was calculated by using the Mosteller formula^21^. Each participant was also fitted with an Actical^®^ omnidirectional accelerometer (Mini-Mitter Co., Inc, Montréal, Québec, Canada) to monitor total energy expenditure (TEE) as a measure of physical activity.

### Acute mental stress hemodynamic testing

All participants were in a fasting state during psychophysiological stress testing. The STROOP-CWC test assesses cognitive interference control in an incongruent manner, requiring participants to identify the colors of color-word cards in contrasting ink colors under time pressure^22^. The STROOP-CWC test was conducted by a trained observer according to CWC protocol, thus the observer maintained a neutral facial expression, rectified incorrect answers, and prompted a faster response. In a semi-recumbent position, beat-to-beat BP changes were continuously assessed throughout psychophysiological stress testing via the Finometer device (Finapres Medical Systems^®^, Amsterdam, The Netherlands) which is validated for assessing beat-to-beat BP variation^19^, and calibrated prior to each measurement. After two minutes, the finger pressure was calibrated with the brachial pressure^23^. To ensure that resting BP values have stabilized, an additional resting period of ten minutes elapsed prior to psychophysiological stress task (STROOP-CWC test) was administered for one minute^22,24^. The average of the last three minutes of the resting recordings and the average of the last 20 to 30 s of the stressor recordings were used in data analyses as this reflects a true baseline. This specific window was selected as being where the exposure measures were evident at a stable plateau value and thus negating the influence of anticipation stress. An integrated age-dependent aortic flow curve was calculated from the surface area beneath the pressure/volume curve using the Beat-Scope version 1.1a software package (Finapres Measurement Systems)^19^. This calculation determined stroke volume (SV), cardiac output (CO), total peripheral resistance (TPR), Windkessel arterial compliance (C_wk_), left ventricular ejection fraction (LVEF) and mean arterial pressure (MAP). The percentage change (∆%) in SBP, DBP, TPR, SV, CO, HR and Cwk from resting values to plateau values obtained during application of a laboratory stressor is used to calculate maximal cardiovascular reactivity of each parameter^8,12,14,25^. To increase performance, a monetary incentive was given according to the number of correct answers^24^.

### Characterizing a predominant α- or β-adrenergic reactivity profile

In the current study, we aimed to novelly characterize a predominant adrenergic hemodynamic profile using non-invasive, hemodynamic recordings obtained during acute stress testing. The adrenergic-hemodynamic reactivity profile was determined and used to profile participants’ responses as predominant α-adrenergic (decreases CO and C_wk_) reactivity (lowest quartile)); and β-adrenergic (increases in CO and C_wk_ (highest quartile)) responses. The key motivation for only using CO and C_wk_ in the characterization of the adrenergic responses is due to the combined hemodynamic principles, as an increase in CO would constitute an increase in HR and/or SV (CO = HR x SV) – which are key differences in the α/β adrenergic hemodynamic responses. Additionally, as increases in TPR and C_wk_ is observed in an α-adrenergic response, with the opposite being true for β-adrenergic responses, C_wk_ was used as the second profiling variable – in an attempt to describe the hemodynamic result of a predominant α/β adrenergic hemodynamic response. Thus, the 25th percentile of ∆%CO and ∆%C_wk_ was used to reflect predominant α-adrenergic responders, given the documented decrease in these parameters during acute stress testing, and the 75th percentile of ∆%CO and ∆%Cwk reflected predominant β-adrenergic responders, as increases in these parameters are reported characteristics of this adrenergic response. Importantly, although the Finapress modelflow algorithm relies on secondary calculations, this model is validated for beat-to-beat variation (continuous flow)^23^. Of the 392 participants with complete acute stress hemodynamic reactivity, 48 presented with predominant α-adrenergic and 69 presented with predominant β-adrenergic profiles respectively (*N* = 117). The remaining *n* = 275 participants could be defined as mixed-adrenergic responders, as their hemodynamics did not fall within the two extremes. Yet, the focus of the current study was to define the risk profile based on the two adrenergic extremes, as determined non-invasively.

### Ambulatory blood pressure and heart rate variability

The ABPM and 2-lead ECG monitor device (Cardiotens CE120^®^; Meditech CE120; Meditech, Budapest, Hungary) was removed on the second day of the procedure. Day-and nighttime hypertension were defined, according to the European Society of Hypertension and European Society of Cardiology guidelines, as systolic BP of ≥ 135 mmHg and/or diastolic BP of ≥ 85 mm Hg (daytime) and systolic BP of ≥ 120 mm Hg and/or diastolic BP of ≥ 70 mmHg (nighttime)^26^. Additionally, only participants with an inflation rate of >75% were included in this analyses.

Heart rate variability (HRV) measures including time- and frequency-domain analyses were obtained with the Cardiotens^®^ (Meditech CE120^®^; Meditech, Budapest, Hungary) and calculated using the CardioVisions^®^ (Meditech CE120^®^; Meditech, Budapest, Hungary) software. The software removed all arrhythmias and extra-ventricular beats, and additional outliers were manually removed before frequency- and time-domain variables were calculated^27^. For supportive analyses, HRV variables used included time-domain parameters, i.e., the standard deviation of the NN intervals (SDNN) and the triangular index (HRVti). To ensure reliability and validity of data, only normal R-R peaks were selected. Frequency-domain parameters included the LF/HF ratio. It is important to note that components of HRV provide measurement of the degree of autonomic modulation and tone rather than the level of autonomic nervous system (ANS) tone^27^. Therefore, for the sake of clarity, all references to time-domain parameters will be described in terms of SNS *activity/tone* and references to frequency-domain parameters will indicate SNS *modulation*.

### Ischemic events

Ischemic events were recorded by 24-H Holter ECG and defined based on specific electrocardiographic criteria. An ischemic event was recorded if there was horizontal or descending ST-segment depression of at least 1 mm, with the duration of the ST-segment episode lasting for a minimum of one-minute, and a minimum interval of one-minute from the preceding episode^7,27^. This definition was used to assess silent myocardial ischemia (SMI) during the SABPA study^19^.

### Biochemical analyses

Fasting blood samples were obtained from the antebrachial vein branches of each participant’s dominant arm with a sterile winged infusion set, by a registered nurse. Blood samples were handled according to the standardized protocol and serum and plasma samples were stored at -80˚C until analyzed in duplicate^19,28,29^. Cotinine values were determined by means of a homogeneous immunoassay with an automated Modular system (Roche, Switzerland). Gamma-glutamyl transferase (γGT) and ultra-high sensitivity C-reactive protein (hs-CRP) were analyzed with the enzyme rate method with Unicel DXC 800 (Beckman and Coulter, Germany). Ethylenediaminetetraacetic acid (EDTA) whole blood glycated hemoglobin (HbA1c) was measured via turbidometric inhibition immunoassay (Cobas Integra 400 plus, Roche, Switzerland) and serum total cholesterol and high-density lipoprotein (HDL) cholesterol were determined with a timed-end-point method, Unicel DXC 800 (Beckman and Coulter, Germany). The cholesterol-to-HDL ratio (total cholesterol: HDL) was calculated by dividing total cholesterol by HDL-cholesterol. Amino-terminal pro-B-type natriuretic peptide (NT-proBNP) and cardiac troponin-T (cTnT) were measured via a high sensitivity electrochemiluminecence method on the Roche^®^ e411 (Roche^®^, Basel, Switzerland). Inter-batch variability 4.6%; intra-batch variability 4.2%. Below detectable limit cTnT values (31.3% of all cTnT analyses in the larger study, *n* = 98), were logarithmically calculated. Additionally, we applied cTnT ≥ 4.2ng/mL for the total group^7^, indicating increased cardiac stress related to cardiovascular risk. Abnormal glucose tolerance, which is a composite term that includes prediabetes and diabetes, was defined at a HbA1c  ≥ 5.7 and/or fasting plasma glucose >100mg/dL and/or diabetes medication usage ^28,29^.

### Stroke risk indicator

The 10-year University of California (UCLA) risk composite score included sex, SBP, hypertensive drugs, diabetes, smoking habit, perfusion deficits, atrial fibrillation and electrocardiography (ECG) left ventricular hypertrophy (American Heart and Stroke certified UCLA Medical Centre, Primary Stroke Centre, Santa Monica, Los Angeles, USA). Medium-to-high stroke probability was defined by scores of 5.2 and greater^6^.

### Statistical analyses

IBM^®^ SPSS^®^ Statistics version 29 software (IBM Corporation; Armonk, New York, USA) was used for data analyses. Kolmogorov-Smirnov tests assessed normality of all variables. The natural logarithm of γGT, hs-CRP, cotinine and HbA1c levels were used in adjusted models. Characteristics between adrenergic responder groups were calculated with *t*-tests. Chi-square (*X*^*2*^) statistics were used to determine proportions and prevalence data. In basic comparisons, HRV parameters were adjusted for age, sex, and ethnicity. A priori covariates included age, sex, ethnicity, WC, γGT, cotinine, HOMA-IR, and MAP. Adjusted logistic regression analyses were used to determine odds ratios (OR) (95% confidence intervals (CI)), in several models to determine the probability of a specific adrenergic response profile identifying a more adverse cardiometabolic profile. Therefore, the following probabilities were calculated: the 10-year stroke risk probability (UCLA stroke risk score); ischemic events; cardiac stress (cTnT ≥ 4.2ng/mL); 24-hour hypertension; low HDL-cholesterol levels; increased central adiposity (WC) and Abnl-GT (combined prediabetes and diabetes).

## Results

Interaction testing revealed that significant differences existed between predominant α- or β-adrenergic responders, independent of a priori selected covariates for cTnT (F_1, 117_ =24.2, *P* < 0.001), NT-proBNP (F_1, 117_ =11.57, *P* = 0.014), 24-hour hypertension (%) (F_1, 117_ =27.89, *P* < 0.001) and 10-year stroke risk probability (%) (F_1, 117_ =11.67, *P* = 0.003). Justifying comparative analyses between these two predominant adrenergic reactivity groups.

### Baseline characteristics

In Table 1 the basic characteristics according to predominant adrenergic profile revealed that the α-adrenergic group were older, consisted of more men and Black Africans (*P* < 0.001), had lower HDL-cholesterol (*P* = 0.041), and higher WC (*P* = 0.005), physical activity (*P* < 0.001), levels of γGT (*P* < 0.001), hs-CRP (*P* = 0.008), NT-proBNP (*P* = 0.046) and insulin (*P* = 0.002). Predominant α-adrenergic responders also had a higher prevalence of insulin resistance (HOMA-IR) (*P* = 0.007), hypertension (*P* < 0.001) and Abnl-GT (*P* < 0.001) compared to the predominant β-adrenergic responders. Whereas the β-adrenergic responders had a higher prevalence of ischemic events (*P* = 0.009). Predominant α-adrenergic responders showed signs of reduced HRV, with lower SDNN (*P* < 0.001), HRVti (*P* < 0.001) and a higher LF/HF ratio (*P* = 0.047) compared to the predominant β-responders.

**Table 1:** Basic characteristics comparing predominant alpha- and beta-adrenergic responders (*N* = 117).

Variable		α-adrenergic responders (*n* = 48)		β-adrenergic responders (*n* = 69)		*P*-value	
Demographic and lifestyle parameters							
Age, years		49 ± 8		41 ± 10		0.001	
Sex, n (% Men)		23 (47)		29 (42)		0.192	
Ethnicity, n (% Black African)		30 (63)		17 (29)		< 0.001	
Body surface area (m^2^)		1.87 ± 0.24		1.91 ± 0.29		0.142	
Waist circumference (cm)		100.14 ± 16.56		90.01 ± 17.05		0.005	
Physical activity (kcal/day)		3294.02 ± 948.13		2709.70 ± 727.00		< 0.001	
*γGT (U/L)		67.1 (38.22; 181.55)		25.71 (19.21; 42.58)		< 0.001	
*Cotinine (ng/mL)		38.67 (11.28; 116.85)		21.32 (10.19, 99.64)		0.921	
CWC baseline hemodynamic parameters							
SBP (mmHg)		144 ± 10		132 ± 11		< 0.001	
DBP (mmHg)		91 ± 8		85 ± 10		< 0.001	
HR (beats/min)		68 ± 12		62 ± 10		0.035	
SV (mL)		114.5 ± 33.2		92.9 ± 28.5		0.002	
CO (L/min)		6.7 ± 3.7		7.5 ± 3.4		0.001	
TPR (mmHg/mL/s)		1.03 ± 0.02		0.98 ± 0.07		0.047	
Cwk (mL/mmHg)		2.02 ± 0.24		3.32 ± 0.07		< 0.001	
LVEF (s)		0.32 ± 0.01		0.33 ± 0.01		0.189	
Cardiometabolic profile							
hs-CRP (mg/L)		5.3 (3.3; 10.4)		1.5 (1.0; 5.5)		0.008	
*NT-proBNP (pg/mL)		62.9 (26.3; 137.0)		41.1 (27.6; 77.2)		0.046	
cTnT (pg/mL)		5.5 ± 3.8		5.4 ± 3.0		0.969	
HbA1c (%)		5.7 ± 0.9		5.4 ± 0.2		0.053	
Insulin (µU/mL)		11.8 ± 5.9		8.6 ± 3.1		0.002	
*HOMA-IR		2.85 (1.75, 5.59)		2.02 (1.58, 3.11)		0.007	
Total cholesterol (mmol/L)		5.1 ± 2.0		5.2 ± 1.6		0.682	
Triglycerides (mmol/L)		1.59 ± 0.94		0.98 ± 0.49		< 0.001	
*HDL-cholesterol (mmol/L)		1.05 (0.78; 1.36)		1.22 ± (1.02; 2.37)		0.041	
Total cholesterol: HDL		5.21 ± 1.73		4.72 ± 1.57		0.048	
Ambulatory blood pressure and HRV profile							
24-hour SBP (mmHg)		139 ± 13		127 ± 10		< 0.001	
24-hour DBP (mmHg)		87 ± 9		82 ± 8		< 0.001	
24-hour MAP (mmHg)		104 ± 9		94 ± 8		< 0.001	
^#^SDNN		101.86 ± 36.54		152.40 ± 48.00		< 0.001	
^#^HRVti		29.23 ± 11.92		37.01 ± 11.24		< 0.001	
^#^LF/HF		3.78 ± 2.01		3.17 ± 2.04		0.047	
Medical history and risk							
Increased 10-year stroke risk		5.6 ± 3.1		2.8 ± 2.1		0.012	
Ischemic events, n (%)		21 (44)		35 (51)		0.009	
Hypertensive medication, n (%)		14 (29)		5 (7)		0.004	
ACE-inhibitors, n (%)		4 (8)		2 (3)		0.078	
Angiotensin II antagonists and receptor blockers, n (%)		1 (2)		1 (1)		0.985	
Thiazide diuretics, n (%)		6 (13)		1 (1)		0.021	
Calcium channel blockers, n (%)		3 (6)		1 (1)		0.097	
Hypertension status, n (%)		38 (77)		23 (33)		< 0.001	
Diabetes medication, n (%)		10 (20)		5 (7)		0.001	
Oral diabetes medication		7 (15)		3 (4)		0.006	
Using insulin for diabetes		3 (6)		2 (3)		0.067	
Abnormal glucose tolerance, n (%)		35 (71)		25 (37)		< 0.001	

Data presented as mean ± SD.

Hypertension status determined by medication usage as well as undiagnosed hypertension through Ambulatory blood pressure measurements.

Abnormal glucose tolerance status determined by diabetic medication usage as well as undiagnosed ABnl-GT by HbA1c ≥ 5.7 and or fasting blood glucose > 100 mg/dL.

*α* alpha, *β* beta, *CO* cardiac output, *cTnT* cardiac troponin-T, *CWC* color-word-conflict, *Cwk* windkessel arterial compliance, *DBP* diastolic blood pressure, *γGT* gamma-glutamyl transferase, *LF/HF* low frequency-high frequency band ratio, *MAP* mean arterial pressure, *HbA1c* glycated hemoglobin, *HDL* high-density lipoprotein, *HOMA-IR* homeostatic model assessment for insulin resistance, *HR* heart rate, *HRV* heart rate variability, *HRVti* triangular index, *hs-CRP* high-sensitivity C-reactive protein, *NT-proBNP* amino-terminal pro-B-type natriuretic peptide, *SBP* systolic blood pressure, *SDNN* standard deviation of the NN intervals, *SV* stroke volume, *TPR* total peripheral resistance.

*Data presented as median (lower and upper quartile).

^#^HRV adjusted for age, sex and ethnicity.

Adjusted reactivity parameters (i.e. acute hemodynamic changes in BP, SV, CO, TPR and C_wk_) indicated that significant differences were maintained (all *P* < 0.001), even after adjustments for age, sex, ethnicity, WC, γGT, cotinine and MAP (Table 2). Importantly, the main hemodynamic reactivity parameters (SBP, DBP, CO, SV, TPR, C_wk_) remained comparable even when assessed within ethnic-specific adrenergic responder groups (Table S1). However, due to limited statistical power, the groups were not further sub-divided into sex and ethnicity. Additional analyses also indicate the adjusted reactivity parameters within the mixed-adrenergic group (Tables S2 and S3).

**Table 2:** Adjusted comparisons between predominant alpha- and beta-adrenergic responders (*N* = 117).

Variable	α-adrenergic responders (*n* = 48)	β-adrenergic responders (*n* = 69)	*P*-value
CWC % change in hemodynamic parameters			
%ΔSBP	17 ± 6	13 ± 7	< 0.001
%ΔDBP	24 ± 7	9 ± 4	< 0.001
%ΔHR	16 ± 10	40 ± 9	< 0.001
%ΔSV	−22 ± 12	6 ± 8	< 0.001
%ΔCO	−4 ± 8	44 ± 11	< 0.001
%ΔTPR	29 ± 5	−24 ± 8	< 0.001
%ΔCwk	−24 ± 9	-4 ± 9	< 0.001
Cardiometabolic profile			
*hs-CRP (mg/L)	6.28 (3.72; 16.11)	4.45 (2.02; 11.21)	0.012
*NT-proBNP (pg/mL)	62.87 (26.27; 136.95)	41.12 (27.55; 77.17)	0.046
cTnT (pg/mL)	5.52 ± 2.56	5.44 ± 3.04	0.969
HbA1c (%)	5.88 ± 0.98	5.51 ± 0.39	0.048
Insulin (µU/mL)	18.02 ± 0.06	11.59 ± 2.95	0.002
HOMA-IR	4.85 ± 2.09	2.25 ± 3.89	< 0.001
Total cholesterol (mmol/L)	5.05 ± 1.11	5.24 ± 1.31	0.262
Triglycerides (mmol/L)	1.59 ± 0.94	1.05 ± 0.49	< 0.001
*HDL-cholesterol (mmol/L)	1.05 (0.78; 1.36)	1.20 ± (1.02; 2.37)	0.041
Total cholesterol: HDL	5.21 ± 1.73	4.72 ± 1.57	0.048

All analyses adjusted for age, sex, ethnicity, waist circumference, gamma-glutamyl transferase, cotinine and mean arterial pressure.

*α* alpha, *β* beta, *CO* cardiac output, *cTnT* cardiac troponin-T, *CWC* color-word-conflict, *Cwk* Windkessel arterial compliance, *DBP* diastolic blood pressure, *HbA1c* glycated hemoglobin, *HDL* high-density lipoprotein, *HOMA-IR* homeostatic model assessment for insulin resistance, *HR* heart rate, *hs-CRP* high-sensitivity C-reactive protein, *NT-proBNP* amino-terminal pro-B-type natriuretic peptide, *SBP* systolic blood pressure, *SV* stroke volume, *TPR* total peripheral resistance.

*Data expressed as median (inter quartile ranges).

### Odds for increased cardiovascular risk

**Fig. 2:**
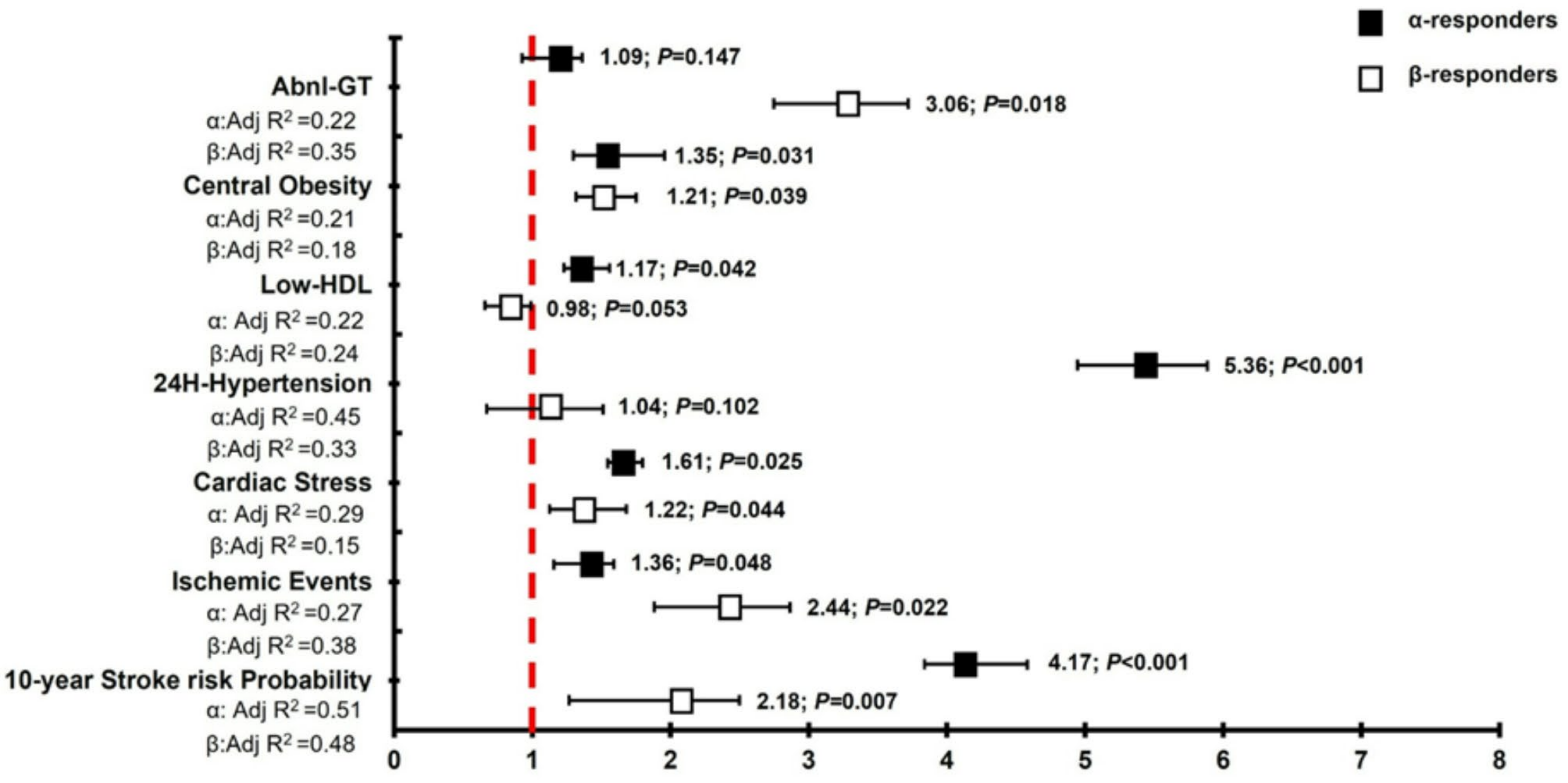
Odds ratios to determine the probability of a specific adrenergic response profile predicting a more adverse cardiometabolic outcome. The following probabilities were calculated: the 10-year stroke risk probability (UCLA stroke risk score ≥ 5.2); 24 h ischemic events; cardiac stress (cTnT ≥ 4.2ng/mL); 24 h hypertension; low HDL-cholesterol levels; increased central adiposity (waist circumference) and abnormal glucose tolerance (Abnl-GT; combined prediabetes and diabetes).

Figure 2 illustrates the adjusted odds within each reactivity profile, identifying adverse cardiometabolic outcomes. The predominant α-adrenergic pattern identified a medium-to-high 10-year stroke probability risk [OR 4.17 (95% CI 2.74; 6.06) *P* < 0.001], 24-hour ischemic events [OR 1.36 (1.19, 1.58) *P* = 0.048), increased cardiac stress [OR 1.61 (1.35, 1.97) *P* = 0.025], likelihood of 24-H hypertension [OR 5.36 (4.05, 6.79) *P* < 0.001], low HDL-cholesterol [OR 1.17 (1.08, 1.24) *P* = 0.042] and greater WC [OR 1.35 (1.12, 1.58] *P* = 0.031]. The predominant β-responders showed significant odds of medium-to-high stroke risk [OR 2.18 (1.95, 2.36) (*P* = 0.007)], cardiac stress [OR 1.22 (1.05, 1.42), *P* = 0.044), central adiposity [OR 1.21 (1.03, 1.48) *P* = 0.039], and even greater odds of 24-H ischemic events (*P* = 0.022) and Abnl-GT (*P* = 0.018), when compared to the predominant α-responders. The mixed-adrenergic group exhibited no significant risk factors apart from 24-H hypertension and increased central obesity (Table S4).

### Sensitivity analyses

We conducted adjusted odds ratio analyses with the same set of covariates within models in the predominant adrenergic responders’ groups. Analyses were repeated by excluding any participant on hypertensive medication (*n* = 19) or anti-diabetic medication (*N* = 15). None of these medications showed a statistically significant impact on the outcome. Therefore, these participants were included in all analyses, as exclusion would have further reduced the sample size.

## Discussion

To our knowledge, this is the first study to characterize and profile the cardiometabolic risk related to a specific acute mental stress-induced adrenergic response – either predominantly α-or β-adrenergic. We showed that, independent of age, sex and ethnicity, both α-and β-adrenergic responders had distinct cardiometabolic risk profiles where the α-adrenergic responders may have a more pressure, cardiovascular-linked risk profile, compared to the β-adrenergic responders who might have a more metabolically driven risk.

### Risk related to predominant α-adrenergic hemodynamic responders

It has been reported that key hemodynamic characteristics, such as decreased arterial compliance (C_wk_) and increased TPR, are hallmarks of an α-adrenergic response, and has been linked to cardiometabolic risk^13,19,30–32^. Yet these studies did not describe or profile a predominant α-or β-adrenergic response, nor independently linked a particular hemodynamic profile to various different cardiometabolic risk factors and outcomes.

When a SNS hyperactive state is perpetuated, as is observed in sustained stress, the biological affinity of norepinephrine to bind to adrenergic receptors, shifts in favor of peripheral, vascular α_1_-adrenergic receptors, but may also lead to an increase in α_1_-adrenergic receptor responsiveness due to receptor upregulation^17,18^. Such a sustained stress state can shift the balance from β_1_-and β_2_-adrenergic signaling in favor of α_1_-mediated effects on vascular tone and resistance. The dominance of SNS tone and modulation observed in the predominant α-adrenergic response group, reflected by depressed HRV, may support this notion. Importantly, HRV does not directly reflect SNS activation or activity, but may reflect SNS tone and modulation^27^. Also, reduced HRV has been linked to decreased β_1_-receptor activation and β_1_-receptor density in the central cardiac tissue^33^, further supporting the predominance of α-adrenergic receptor activation. This state of SNS hyperactivity contributes to the propagation of various cardiometabolic adversities. The preference for α-adrenergic receptor activation, and thus an overall decrease in CO and arterial compliance, specifically during stressful conditions that require additional perfusion, may have detrimental effects. In SNS hyperactivity, a high-pressure state persists, reflected by the significant prevalence of hypertension and risk for hypertension identified in this group, accompanied by increased cardiac stress. This condition may co-exist with and be exacerbated by comorbidities such as low HDL-cholesterol and increased central adiposity – hallmark features of the metabolic syndrome^34^.

The adverse effects of sustained SNS hyperactivation, inducing higher systemic pressure, are also reflected by the significantly increased odds of ischemic events as well as higher stroke risk probability in the predominant α-adrenergic responder group. Where α_1_-receptors (cardiac) ensure ischemic preconditioning and physiological hypertrophy^35^, excessive activation of peripheral α_2_-receptors, resulting in decreased output, increased SBP, increased TPR and decreased arterial compliance increases the risk of peripheral perfusion deficits. Additionally, α_2_-receptor activation also contributes to coronary vasoconstriction^36^. Indeed, when coronary circulation is compromised due to hypertension and SNS hyperactivity, the autoregulatory aspects of the microvessels are impaired, and α-adrenergic mediated vasoconstriction may occur, thereby reducing coronary perfusion, creating a milieu for myocardial ischemia as well as stroke. This is reflected by significant odds of a higher number of ischemic events predicted by an α-adrenergic response. Sympathetic hyperactivity may affect the excitation-contraction coupling and calcium signaling, thereby enhancing the risk for apoptosis and leading to the progression of ischemic heart failure^30^.

The ischemic risk related to excessive SNS activity and activation of an α-adrenergic pattern, is also supported by the higher 10-year stroke risk probability in this group. A shift in the autonomic balance in favor of SNS activity has been linked to increased risk of stroke, as well as poorer outcomes post-stroke^3^. In α-adrenergic responders, this is further supported by the increased odds for higher cTnT, which is a marker of cardiac stress, but has also been related to stroke risk in a South African population^6^.

**Fig. 3:**
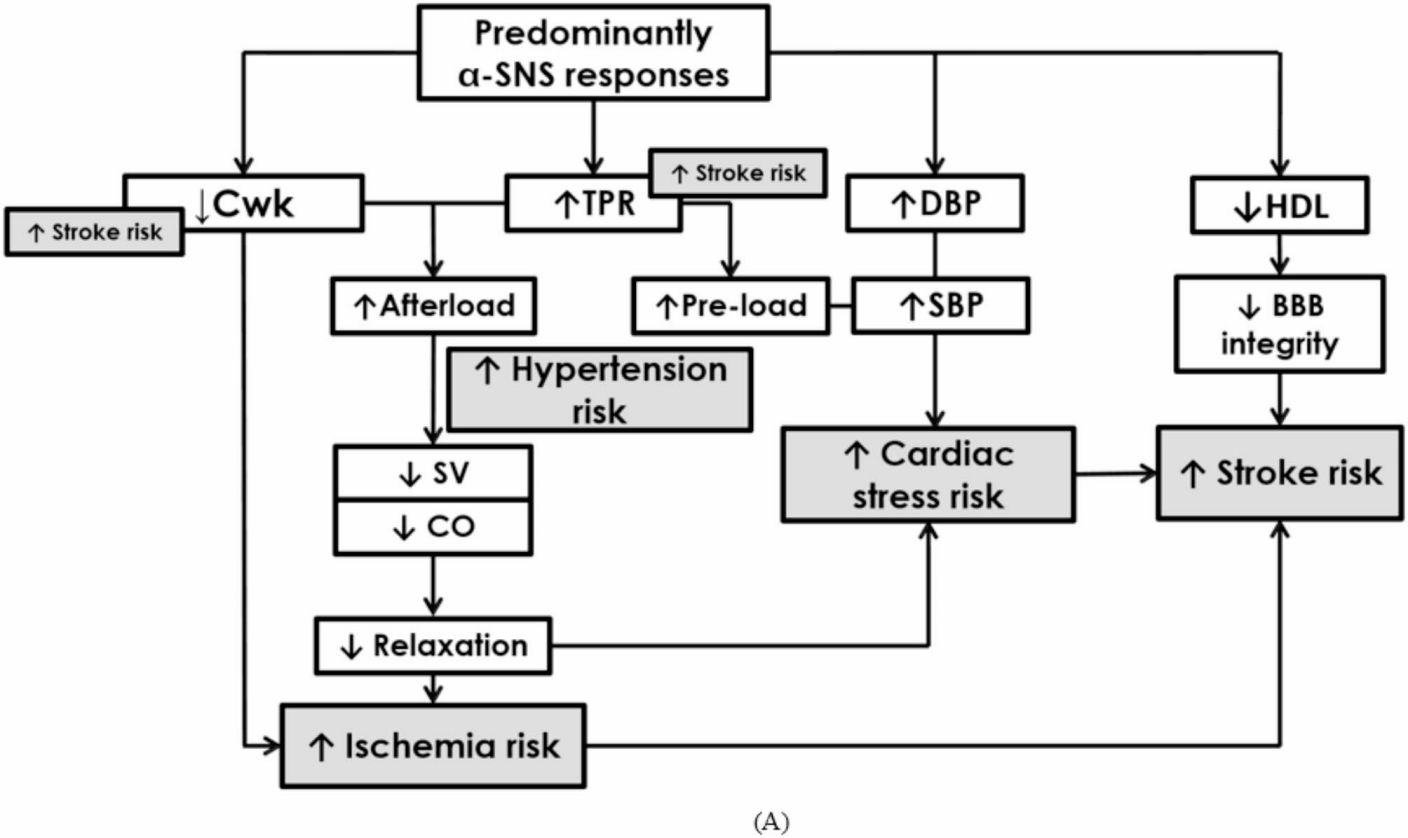

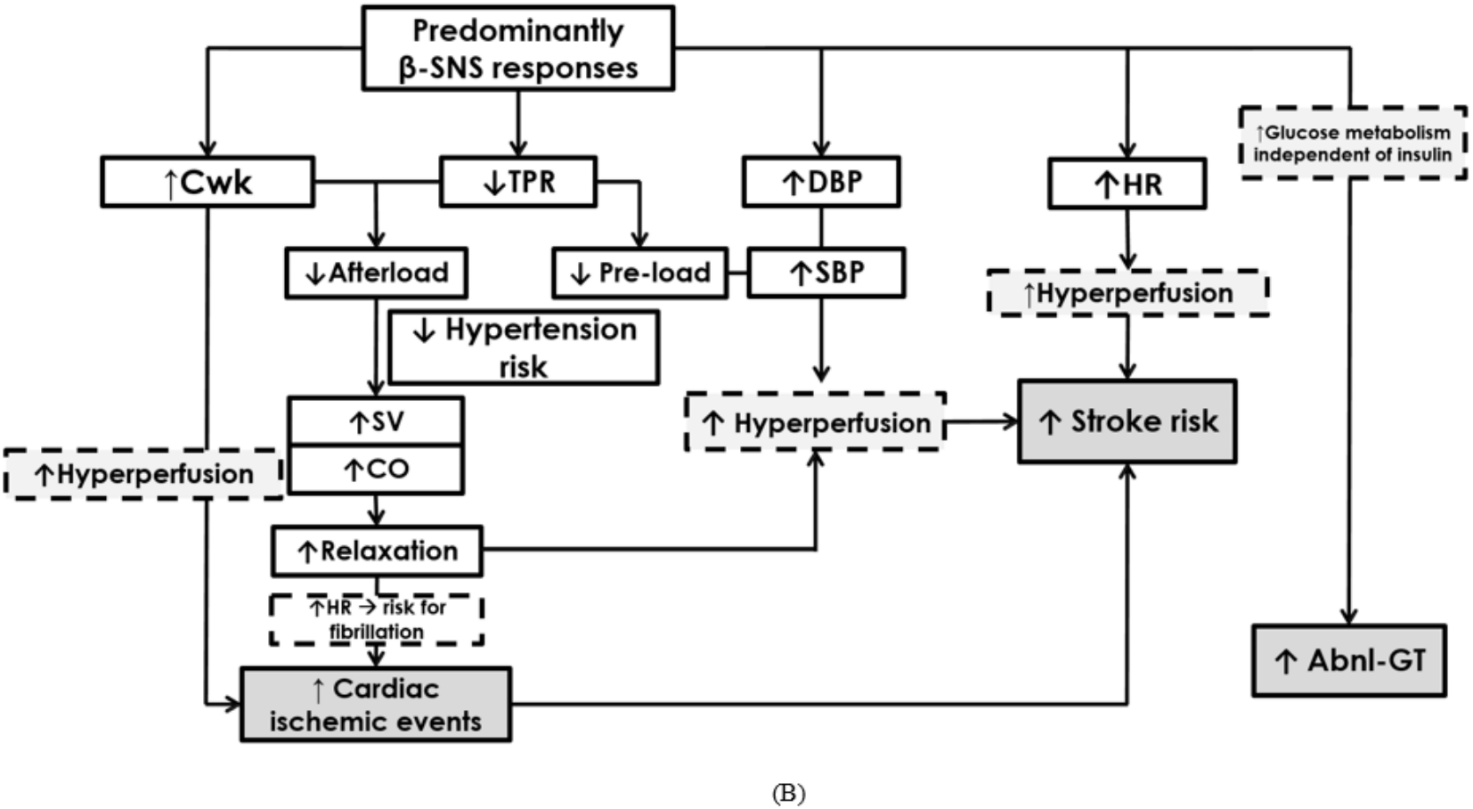
(**A**) Hypothetical mechanisms linking cardiovascular and ischemic risk to a predominant alpha (α)-adrenergic acute stress response. *BBB* blood-brain barrier, *CO* cardiac output; *C*_*wk*_ Windkessel arterial compliance, *DBP* diastolic blood pressure, *HDL* high-density lipoprotein, *SBP* systolic blood pressure, *SNS* sympathetic nervous system, *SV* stroke volume, *TPR* total peripheral resistance. (**B**) Hypothetical mechanisms linking metabolic and ischemic risk to a predominant beta (β)-adrenergic acute stress response. *Abnl-GT* abnormal glucose tolerance, *CO* cardiac output, *C*_*wk*_ Windkessel arterial compliance, *DBP* diastolic blood pressure, *HR* heart rate, *SBP* systolic blood pressure, *SNS* sympathetic nervous system, *SV* stroke volume, *TPR* total peripheral resistance.

We cautiously propose a hypothetical mechanism suggesting that the more adverse cardiovascular profile observed in the α-adrenergic responders may be due to SNS hyperactivity, shifting the balance in favor of α_1_-adrenergic activity, resulting in a peripheral, high-pressure system. In a predominant α-adrenergic hemodynamic pattern, less compliance (lower Cwk), increased resistance (TPR) and increased DBP are observed. This hemodynamic combination will increase pre-load, thereby corroborating the increased hypertension prevalence and risk observed. The increased DBP reflects increased cardiac strain (reflected by the higher odds of cTnT), which has been shown to be linked to increased stroke risk, independently of other cardiovascular factors^6^. The combination of increased peripheral resistance and decreased arterial compliance contribute to an increased afterload, further evidenced by the decreased SV and CO. This indicates decreased relaxation, further reflecting the increased cardiac strain, but also increased risk for ischemic events and subsequent stroke risk. This represents a pressure, cardiovascular-centered risk profile and these hypothetical events are depicted in (Fig. 3A).

This may indicate that in a sustained SNS hyperactive state, an α-adrenergic hemodynamic response may be considered as a risk factor for cardiovascular disease (CVD) in itself; however, analyses indicating the relationship between the catecholamines and specific hemodynamic parameters, as well as longitudinal analyses will be required to verify and expand on this hypothesis.

### Risk related to predominant β-adrenergic hemodynamic responders

In comparison to the α-adrenergic responders, predominant β-adrenergic responders present with a less prominent adverse cardiometabolic profile, with a lower prevalence of hypertension, Abnl-GT, and a lower stroke risk. Yet, where the α-adrenergic responders may have a more pressure, cardiovascular-linked risk profile, the β-adrenergic responders might have a more metabolically driven risk. Initially, it may appear that the predominant β-adrenergic responders show a small CVD risk. However, there are significant odds of 10-year stroke risk, ischemic injury, and Abnl-GT in this group. It is important to consider that with an exaggerated and sustained β-adrenergic response, there might be the risk of hyperperfusion injuries, ischemia and increased oxidative strain^37^.

During stressful events, β-adrenergic receptors are activated, increasing contractility, rate of contractility and strength of cardiomyocyte contraction. If a stressor is mismanaged, and the demand exceeds the supply, myocardial ischemia can occur^16,18,38^. Upon restoration of blood flow, especially after such a brief period of ischemia, β-adrenergic activation may exacerbate reperfusion injury^39^. The sudden reintroduction of oxygen can lead to oxidative stress, local inflammation and cardiomyocyte damage, particularly when β-adrenergic receptors are persistently stimulated, it can promote cardiomyocyte apoptosis and eventual maladaptive remodeling of the cardiac tissue^39^. This might support the increased odds for cardiac stress, as reflected by cTnT levels greater than 4.2 ng/mL. Oxidative stress can damage endothelial cells and impair vascular function, complicating future responses to ischemia, potentially leading to hyperperfusion once blood flow is restored. Yet this proposed sequence of events requires further investigation.

Similar mechanisms may be involved with the significant link to 10-year stroke risk in this group. During conditions of perpetual β-adrenergic activation, specifically β_1_-adrenergic receptors, the vasodilatory responses of the cerebral arteries may be impaired^16^, which may relate to an increased risk of stroke partly due to the dysregulation in cerebrovascular tone. Similarly, excessive β-adrenergic signaling can increase oxidative stress, and enhance the vascular inflammatory responses, contributing to vascular inflammation and atherogenesis, both risk factors in stroke^40^.

Surprisingly, the odds for Abnl-GT were only significant in the β-adrenergic responders, despite less participants with Abnl-GT. There are several hypothetical mechanisms through which excessive β-adrenergic activation can contribute to the increased likelihood of Abnl-GT observed in the predominant β-adrenergic responders. Given that the β-adrenergic responders did not show significant insulin resistance in comparison to the α-adrenergic responders, it can be postulated that sustained β-adrenergic activation may impact glucose metabolism via insulin-independent mechanisms^38,41^. Excessive β-adrenergic activation directly impacts glucose transporters (GLUT), GLUT1 and GLUT4, via both β_1_- and β_2_-adrenergic receptor activation^38^. β-adrenergic receptor activation enhances glucose uptake in skeletal muscle and adipose tissue by modulating the activity of GLUT. β-adrenergic, particularly β_2_, receptors can increase the activity of GLUT1 even in the absence of insulin signaling^42^. Similarly, β-adrenergic activation increases cyclic adenosine monophosphate (cAMP) which can promote glucose metabolism independently from insulin^38^ and may shift the metabolism from glucose to fatty-acids, especially under conditions of stress. This supports as during increased central cardiac activity, cardiac tissue utilized fatty and lipid metabolism in favor of glucose^43^.

We tentatively suggest a hypothetical mechanism according to which the adverse cardiometabolic profile evident in the β-adrenergic responders relates to metabolic risk and hyperperfusion injury (Fig. 3B).

Although this was not the aim of current study, supplemental analyses in the mixed-adrenergic group indicated an increased odds for 24 H-hypertension and greater visceral adiposity, yet no other risk factors were significant (Table S4). The significant risk observed in this group might be attributed to its size and the higher number of participants with 24 H-hypertension and increased WC compared to the predominant α-and β-adrenergic responder groups. This may suggest a distinct risk profile associated with each adrenergic extreme, rather than with mixed-adrenergic responders. Thus, increased cardiometabolic risk overall may be linked to a predominant adrenergic profile, with each profile indicating a unique risk. Additional analyses investigating the spectrum of α-and β-adrenergic hemodynamic characteristics in this mixed-responder group is required, to gain a better understanding of the distinct hemodynamic patterns and predominance within this sub-group – yet this was beyond the scope of the current investigation.

### Strengths, limitations and future directions

Despite our small sample size, the strength of the current studies lies in the rigorous sampling, controlled research environment, stress testing protocol and study population. Our teacher-cohort had similar socioeconomic and educational status, similar access to healthcare and were from a similar working environment. Acute stress hemodynamic responses were measured on a single occasion via the validated Finometer device for beat-to-beat BP variation. This was the first study to attempt characterization of a predominant adrenergic profile using only non-invasive hemodynamic assessment via Finometer beat-to-beat BP and hemodynamic variation. Longitudinal risk assessment, including central hemodynamic reactivity measured via echocardiogram (Table S5), should be conducted to draw mechanistic conclusions, yet our study provides the basis for such longitudinal analyses. Our sample size was limited; therefore, the study was exploratory in nature, and future studies may compare larger groups based on specific clinical outcomes and pharmacological treatment. Given that our sample population was from a specific area in South Africa, our findings cannot necessarily be extrapolated to the broader South African population, or globally. Yet the preliminary data gathered here can be assessed and tested in larger, more diverse populations. Future studies might consider assessing catecholamines and their relationship with specific hemodynamic parameters within each of these reactivity groups, also investigating the associations within the mixed-adrenergic group, as well as additional mechanisms that might link to the risk identified, such as oxidative stress and acute oxidative stress responses. It would also be prudent to verify the risks linked to the adrenergic-hemodynamic profile identified with tissue sampling and receptor docking studies.

## Conclusion

This was the first study to examine cardiometabolic risk based on a predominant adrenergic profile during acute mental stress application, independent of age, sex and ethnicity. The risk profile identified in predominantly α-adrenergic responders mainly involved the effects of a high-pressure system, cardiac stress, and ischemia, possibly attributed to sustained SNS activity. However, in predominantly β-adrenergic responders, the risk profile may rather be metabolic, hyperperfusion injury related risk. This study describes a unique risk profile related to specific acute adrenergic-hemodynamic response patterns independent of age, sex and ethnicity, indicating that a specific, predominant adrenergic response pattern may be an independent and unique cardiometabolic risk factor.

## Electronic supplementary material

Below is the link to the electronic supplementary material.

Supplementary Material 1

Supplementary Material 2

Supplementary Material 3

Supplementary Material 4

Supplementary Material 5

## Data Availability

All inquiries regarding data availability can be made upon reasonable request to the corresponding author, AW.
