# Supplementary material for: Acute mental stress-induced alpha or beta-adrenergic reactivity patterns linked to unique cardiometabolic risk profiles
Source: Sci Rep. 2025 Mar 13;15:8668. doi: 10.1038/s41598-025-92961-2 (PMC11906893; doi:10.1038/s41598-025-92961-2)
Supplement: Supplementary file 1 — Supplementary Material 1 [file 41598_2025_92961_MOESM1_ESM.docx]

**Table S1: Adjusted comparisons between predominant alpha- and beta-adrenergic responders within each ethnic group (N=117)**

| **Variable** | **α-adrenergic responders (n=48)** | | | | | **β-adrenergic responders (n=69)** | | | |
| --- | --- | --- | --- | --- | --- | --- | --- | --- | --- |
|  | **Black**  **(n=30)** | | **White**  **(n=18)** | | ***P*-value** | **Black**  **(n=17)** | | **White**  **(n=52)** | ***P*-value** |
| *CWC % change in hemodynamic parameters* | | | | | | | | | |
| %ΔSBP | 16±6 | 18±5 | | 0.086 | | 12±8 | 13±4 | | 0.662 |
| %ΔDBP | 21±7 | 22±8 | | 0.325 | | 15±6 | 13±5 | | 0.089 |
| %ΔHR | 17±10 | 19±11 | | 0.069 | | 38±9 | 42±8 | | 0.041 |
| %ΔSV | -23±12 | -21±12 | | 0.097 | | 5±8 | 8±5 | | 0.103 |
| %ΔCO | -6±8 | -5±7 | | 0.182 | | 42±11 | 46±10 | | 0.384 |
| %ΔTPR | 30±4 | 28±6 | | 0.059 | | -23±8 | -26±9 | | 0.093 |
| %ΔCwk | -25±9 | -24±7 | | 0.122 | | -5±8 | -4±6 | | 0.358 |
| *Cardiometabolic profile* | | | | | | | | | |
| *hs-CRP (mg/L) | 6.58 (3.92; 16.93) | 5.95 (3.51;14.32) | | 0.108 | | 4.15 (2.25; 13.32) | 3.78 (1.84; 8.56) | | 0.028 |
| *NT-proBNP (pg/mL) | 60.13 (24.22; 128.41) | 65.74 (31.06; 139.85) | | 0.091 | | 45.08 (28.12; 78.23) | 39.38 (25.31; 65.28) | | 0.065 |
| cTnT (pg/mL) | 5.79 ± 2.75 | 4.36±2.31 | | 0.114 | | 5.65 ± 3.11 | 3.69±2.59 | | 0.024 |
| HbA1c (%) | 5.65 ± 0.95 | 5.73±1.02 | | 0.238 | | 5.49 ± 0.35 | 5.53±0.65 | | 0.284 |
| Insulin (μU/mL) | 17.87 ± 0.09 | 18.13±1.13 | | 0.326 | | 10.94 ± 2.42 | 11.98±3.21 | | 0.118 |
| HOMA-IR | 4.8± 2.1 | 5.0±2.0 | | 0.064 | | 2.2 ± 3.7 | 2.3±3.0 | | 0.092 |
| Total cholesterol (mmol/L) | 5.12 ± 1.10 | 4.91±1.15 | | 0.033 | | 5.36 ± 1.23 | 5.07±1.35 | | 0.046 |
| Triglycerides (mmol/L) | 1.69 ± 0.90 | 1.48±1.01 | | 0.027 | | 1.10 ± 0.47 | 0.97±0.51 | | 0.087 |
| HDL-cholesterol (mmol/L) | 0.98 ±1.35 | 1.11±1.02 | | 0.009 | | 1.18 ± 1.11 | 2.14±1.08 | | 0.014 |
| Total cholesterol:HDL | 5.22 ± 1.47 | 4.42±0.97 | | 0.018 | | 4.54 ± 1.08 | 2.37±1.25 | | 0.001 |

All analyses adjusted for age, sex, waist circumference and mean arterial pressure.

*Data reported as median (interquartile ranges)

Abbreviations: α; alpha; β, beta; CO, cardiac output; cTnT, cardiac troponin-T; CWC, Color-Word-Conflict; Cwk; Windkessel arterial compliance; DBP, diastolic blood pressure; HbA1c, glycated hemoglobin; HDL, high-density lipoprotein; HOMA-IR, homeostatic model assessment for insulin resistance; HR, heart rate; hs-CRP, high-sensitivity C-reactive protein; NT-proBNP, amino-terminal pro-B-type natriuretic peptide; SBP, systolic blood pressure; SV, stroke volume; TPR, total peripheral resistance
