# Supplementary material for: Acute mental stress-induced alpha or beta-adrenergic reactivity patterns linked to unique cardiometabolic risk profiles
Source: Sci Rep. 2025 Mar 13;15:8668. doi: 10.1038/s41598-025-92961-2 (PMC11906893; doi:10.1038/s41598-025-92961-2)
Supplement: Supplementary file 2 — Supplementary Material 2 [file 41598_2025_92961_MOESM2_ESM.docx]

**Table S2: Basic characteristics predominant alpha- and beta-adrenergic and mixed adrenergic responders (N=372)**

| **Variable** | | **α-adrenergic responders (n=48)** | | **Mixed adrenergic responders (n=275)** | | **β-adrenergic responders (n=69)** | | ***P for linear trend*** | |
| --- | --- | --- | --- | --- | --- | --- | --- | --- | --- |
| ***Demographic and lifestyle parameters*** | | | | | | | | | |
| Age, years | | 49 ± 8 | | 45 ± 9 | | 41 ± 10 | | <0.001 | |
| Sex, n (% Men) | | 23 (47) | | 133 (52) | | 29 (42) | | 0.350 | |
| Ethnicity, n (% Black African) | | 30 (63) | | 123 (48) | | 17 (29) | | <0.001 | |
| Body surface area (m^2^) | | 1.87 ± 0.24 | | 1.88 ± 0.31 | | 1.91 ± 0.29 | | 0.269 | |
| Waist circumference (cm) | | 100.14 ± 16.56 | | 94.5 ± 6.08 | | 90.01 ± 17.05 | | 0.007 | |
| Physical activity (kcal/day) | | 3294.02 ± 948.13 | | 3058.34 ± 941.88 | | 2709.70 ± 727.00 | | <0.001 | |
| *γGT (U/L) | | 67.1 (38.22; 181.55) | | 29.6 (27.3, 32.1) | | 25.71 (19.21; 42.58) | | <0.001 | |
| *Cotinine (ng/mL) | | 38.67 (11.28; 116.85) | | 20.69 (12.36; 77.62) | | 21.32 (10.19, 99.64) | | 0.873 | |
| ***CWC Baseline hemodynamic parameters*** | | | | | | | | | |
| SBP (mmHg) | | 144 ± 10 | | 136 ± 9 | | 132 ± 11 | | <0.001 | |
| DBP (mmHg) | | 91 ± 8 | | 79 ± 8 | | 85 ± 10 | | <0.001 | |
| HR (beats/min) | | 68 ± 12 | | 67 ± 8 | | 62 ± 10 | | 0.048 | |
| SV (mL) | | 114.5 ± 33.2 | | 99.8 ± 12.5 | | 92.9 ± 28.5 | | 0.009 | |
| CO (L/min) | | 6.7 ± 3.7 | | 6.69 ± 4.0 | | 7.5 ± 3.4 | | 0.001 | |
| TPR (mmHg/mL/s) | | 1.03 ± 0.02 | | 0.99 ± 0.08 | | 0.98 ± 0.07 | | 0.007 | |
| Cwk (mL/mmHg) | | 2.02 ± 0.24 | | 2.02 ± 0.11 | | 3.32 ± 0.07 | | <0.001 | |
| LVEF (s) | | 0.32 ± 0.01 | | 0.33 ± 0.02 | | 0.33 ± 0.01 | | 0.574 | |
| ***Cardiometabolic profile*** | | | | | | | | | |
| hs-CRP (mg/L) | | 5.3 (3.3; 10.4) | | 2.92 (1.64; 4.69) | | 2.64 (1.0; 5.5) | | <0.001 | |
| *NT-proBNP (pg/mL) | | 62.9 (26.3; 137.0) | | 54.3 (26.9; 80.7) | | 41.1 (27.6; 77.2) | | 0.038 | |
| cTnT (pg/mL) | | 5.5 ± 3.8 | | 5.2 ± 3.2 | | 5.4 ± 3.0 | | 0.269 | |
| HbA1c (%) | | 5.7 ± 0.9 | | 5.7 ± 0.1 | | 5.4 ± 0.2 | | 0.049 | |
| Insulin (μU/mL) | | 11.8 ± 5.9 | | 11.2 ± 4.7 | | 8.6 ± 3.1 | | <0.001 | |
| *HOMA-IR | | 2.85 (1.75, 5.59) | | 2.78 (2.57; 3.02) | | 2.02 (1.58, 3.11) | | 0.012 | |
| Total cholesterol (mmol/L) | | 5.1 ± 2.0 | | 5.1 ± 3.01 | | 5.2 ± 1.6 | | 0.068 | |
| Triglycerides (mmol/L) | | 1.59 ± 0.94 | | 1.09 ± 1.02 | | 0.98 ± 0.49 | | <0.001 | |
| *HDL-cholesterol (mmol/L) | | 1.05 (0.78; 1.36) | | 1.16 (0.85; 1.87) | | 1.22 (1.02; 2.37) | | 0.004 | |
| Total cholesterol:HDL | | 5.21 ± 1.73 | | 4.55 ± 1.46 | | 4.72 ± 1.57 | | 0.013 | |
| ***Ambulatory blood pressure and HRV profile*** | | | | | | | | | |
| 24-hour SBP (mmHg) | | 139 ± 13 | | 128 ± 9 | | 127 ± 10 | | <0.001 | |
| 24-hour DBP (mmHg) | | 87 ± 9 | | 80 ± 9 | | 82 ± 8 | | <0.001 | |
| 24-hour MAP (mmHg) | | 104 ± 9 | | 96 ± 10 | | 94 ± 8 | | <0.001 | |
| ^#^SDNN | | 101.86 ± 36.54 | | 148.31 ± 51.01 | | 152.40 ± 48.00 | | <0.001 | |
| ^#^HRVti | | 29.23 ± 11.92 | | 36.14 ± 10.96 | | 37.01 ± 11.24 | | <0.001 | |
| ^#^LF/HF | | 3.78 ± 2.01 | | 3.20 ±2.06 | | 3.17± 2.04 | | 0.036 | |
| ***Medical history and risk*** | | | | | | | | | |
| Increased 10-year stroke risk | | 5.6 ± 3.1 | | 2.9±2.9 | | 2.8±2.1 | | 0.009 | |
| Ischemic events, n (%) | | 21 (44) | | 10 (3) | | 35 (51) | | <0.001 | |
| Total Hypertensive medication, n (%) | | 14 (29) | | 51 (19) | | 5 (7) | | 0.004 | |
| *ACE-inhibitors, n (%)* | | 4 (8) | | 16 (6) | | 2 (3) | | 0.078 | |
| *Angiotensin II antagonists and receptor blockers, n (%)* | | 1 (2) | | 2 (0.4) | | 1 (1) | | 0.985 | |
| *Thiazide diuretics, n (%)* | | 6 (13) | | 22 (9) | | 1 (1) | | 0.021 | |
| *Calcium channel blockers, n (%)* | | 3 (6) | | 11 (4) | | 1 (1) | | 0.097 | |
| Hypertension status, n (%) | | 38 (77) | | 70 (25) | | 23 (33) | | <0.001 | |
| Total Diabetes medication, n (%) | | 10 (20) | | 20 (7) | | 5 (7) | | 0.041 | |
| *Oral diabetes medication* | | 7 (15) | | 7 (15)n 16 (6) | | 3 (4) | | 0.011 | |
| *Using insulin for diabetes* | | 3 (6) | | 4 (2) | | 2 (3) | | 0.164 | |
| Abnormal glucose tolerance, n (%) | | 35 (71) | | 166 (65) | | 25 (37) | | <0.001 | |

Data presented as mean±SD.

*Data presented as median (lower and upper quartile).

^#^HRV adjusted for age, sex and ethnicity.

Abbreviations: α; alpha; β, beta; CO, cardiac output; cTnT, cardiac troponin-T; CWC, Color-Word-Conflict; Cwk; Windkessel arterial compliance; DBP, diastolic blood pressure; γGT, gamma-glutamyl transferase LF/HF. Low frequency-high frequency band ratio; MAP, mean arterial pressure; HbA1c, glycated hemoglobin; HDL, high-density lipoprotein; HOMA-IR, homeostatic model assessment for insulin resistance; HR, heart rate; HRV, heart rate variability; HRVti, triangular index; hs-CRP, high-sensitivity C-reactive protein; NT-proBNP, amino-terminal pro-B-type natriuretic peptide; SBP, systolic blood pressure; SDNN, standard deviation of the NN intervals; SV, stroke volume; TPR, total peripheral resistance.

Hypertension status determined by medication usage as well as undiagnosed hypertension through Ambulatory blood pressure measurements

Abnormal glucose tolerance status determined by diabetic medication usage as well as undiagnosed ABnl-GT by HbA1c≥5.7 and or fasting blood glucose>100mg/dL.
