# Supplementary material for: Acute mental stress-induced alpha or beta-adrenergic reactivity patterns linked to unique cardiometabolic risk profiles
Source: Sci Rep. 2025 Mar 13;15:8668. doi: 10.1038/s41598-025-92961-2 (PMC11906893; doi:10.1038/s41598-025-92961-2)
Supplement: Supplementary file 3 — Supplementary Material 3 [file 41598_2025_92961_MOESM3_ESM.docx]

**Table S3: Adjusted comparisons between predominant alpha- and beta-adrenergic responders and mixed-adrenergic responders(N=392)**

| **Variable** | **α-adrenergic responders (n=48)** | **Mixed-adrenergic responders**  **(n=275)** | **β-adrenergic responders (n=69)** | ***P*-value for linear trend** |
| --- | --- | --- | --- | --- |
| *CWC % change in hemodynamic parameters* | | | | |
| %ΔSBP | 17±6 | 15±4 | 13±7 | <0.001 |
| %ΔDBP | 24±7 | 13±6 | 9±4 | <0.001 |
| %ΔHR | 16±10 | 30±5 | 40±9 | <0.001 |
| %ΔSV | -22±12 | -7±6 | 6±8 | <0.001 |
| %ΔCO | -4±8 | 24±10 | 44±11 | <0.001 |
| %ΔTPR | 29±5 | -2±6 | -24±8 | <0.001 |
| %ΔCwk | -24±9 | -15±8 | -4±9 | <0.001 |
| *Cardiometabolic profile* | | | | |
| *hs-CRP (mg/L) | 6.28 (3.72; 16.11) | 4.63 (2.68;10.38) | 4.45 (2.02; 11.21) | 0.003 |
| *NT-proBNP (pg/mL) | 62.87 (26.27; 136.95) | 55.69 (25.97; 89.36) | 41.12 (27.55; 77.17) | 0.009 |
| cTnT (pg/mL) | 5.52 ± 2.56 | 5.48±3.09 | 5.44 ± 3.04 | 0.852 |
| HbA1c (%) | 5.88 ± 0.98 | 5.69 ± 0.87 | 5.51 ± 0.39 | 0.021 |
| Insulin (μU/mL) | 18.02 ± 0.06 | 11.02 ±1.68 | 11.59 ± 2.95 | <0.001 |
| HOMA-IR | 4.85 ± 2.09 | 2.80 ± 2.39 | 2.25 ± 3.89 | <0.001 |
| Total cholesterol (mmol/L) | 5.05 ± 1.11 | 5.11 ± 1.56 | 5.24 ± 1.31 | 0.197 |
| Triglycerides (mmol/L) | 1.59 ± 0.94 | 1.10 ± 0.69 | 1.05 ± 0.49 | <0.001 |
| *HDL-cholesterol (mmol/L) | 1.05 (0.78; 1.36) | 1.18 ± 1.02 | 1.20 ± (1.02; 2.37) | 0.059 |
| Total cholesterol:HDL | 5.21 ± 1.73 | 4.55 ± 1.48 | 4.72 ± 1.57 | 0.037 |

All analyses adjusted for age, sex, ethnicity, waist circumference, gamma-glutamyl transferase, cotinine and mean arterial pressure.

*Data expressed as median (interquartile ranges)

Abbreviations: α; alpha; β, beta; CO, cardiac output; cTnT, cardiac troponin-T; CWC, Color-Word-Conflict; Cwk; Windkessel arterial compliance; DBP, diastolic blood pressure; HbA1c, glycated hemoglobin; HDL, high-density lipoprotein; HOMA-IR, homeostatic model assessment for insulin resistance; HR, heart rate; hs-CRP, high-sensitivity C-reactive protein; NT-proBNP, amino-terminal pro-B-type natriuretic peptide; SBP, systolic blood pressure; SV, stroke volume; TPR, total peripheral resistance
