# Supplementary material for: Acute mental stress-induced alpha or beta-adrenergic reactivity patterns linked to unique cardiometabolic risk profiles
Source: Sci Rep. 2025 Mar 13;15:8668. doi: 10.1038/s41598-025-92961-2 (PMC11906893; doi:10.1038/s41598-025-92961-2)
Supplement: Supplementary file 4 — Supplementary Material 4 [file 41598_2025_92961_MOESM4_ESM.docx]

**Table S4 -Supplementary odds ratios in the mixed adrenergic responder group**

**Odds ratio for mixed-adrenergic responders predicting 10-year stroke risk probability**

|  |  | **95% Confidence interval** | | **P-value** |
| --- | --- | --- | --- | --- |
|  | **OR** | **Lower** | **Upper** |  |
| **Adj R^2^: 0.03** | | | | |
| 10-year stroke risk | 0.98 | -1.02 | 3.69 | 0.897 |

**Odds ratio for mixed-adrenergic responders predicting Ischemic events**

|  |  | **95% Confidence interval** | | **P-value** |
| --- | --- | --- | --- | --- |
|  | **OR** | **Lower** | **Upper** |  |
| **Adj R^2^: <0.01** | | | | |
| Ischemic events | 1.06 | -2.67 | 1.47 | 0.631 |

**Odds ratio for mixed-adrenergic responders predicting Cardiac stress**

|  |  | **95% Confidence interval** | | **P-value** |
| --- | --- | --- | --- | --- |
|  | **OR** | **Lower** | **Upper** |  |
| **Adj R^2^: 0.09** | | | | |
| Cardiac stress | 1.65 | 0.54 | 2.11 | 0.352 |

**Odds ratio for mixed-adrenergic responders predicting 24H-Hypertension**

|  |  | **95% Confidence interval** | | **P-value** |
| --- | --- | --- | --- | --- |
|  | **OR** | **Lower** | **Upper** |  |
| **Adj R^2^: 0.18** | | | | |
| 24-H hypertension | 2.56 | 1.59 | 3.64 | 0.041 |

**Odds ratio for mixed-adrenergic responders predicting Low-HDL**

|  |  | **95% Confidence interval** | | **P-value** |
| --- | --- | --- | --- | --- |
|  | **OR** | **Lower** | **Upper** |  |
| **Adj R^2^: <0.01** | | | | |
| Low HDL | -1.33 | -3.69 | 2.87 | 0.473 |

**Odds ratio for mixed-adrenergic responders predicting Central Obesity**

|  |  | **95% Confidence interval** | | **P-value** |
| --- | --- | --- | --- | --- |
|  | **OR** | **Lower** | **Upper** |  |
| **Adj R^2^: 0.23** | | | | |
| Central Obesity | 1.46 | 1.15 | 1.89 | 0.008 |

**Odds ratio for mixed-adrenergic responders predicting Abnl-GT**

|  |  | **95% Confidence interval** | | **P-value** |
| --- | --- | --- | --- | --- |
|  | **OR** | **Lower** | **Upper** |  |
| **Adj R^2^: 0.13** | | | | |
| Abnl-GT | 1.36 | 0.54 | 1.94 | 0.265 |
