# Supplementary material for: Acute mental stress-induced alpha or beta-adrenergic reactivity patterns linked to unique cardiometabolic risk profiles
Source: Sci Rep. 2025 Mar 13;15:8668. doi: 10.1038/s41598-025-92961-2 (PMC11906893; doi:10.1038/s41598-025-92961-2)
Supplement: Supplementary file 5 — Supplementary Material 5 [file 41598_2025_92961_MOESM5_ESM.docx]

**Table S5: Methodological Comparison of Finapress and Echocardiography to measure cardiac output**

| **Feature** | **Finapres (Modelflow)** | **Echocardiography (Doppler)** |
| --- | --- | --- |
| **Algorithm** | Non-linear three-element model of aortic input impedance, using arterial blood pressure (ABP) waveform | Measures left ventricular outflow tract (LVOT) area and velocity time integral (VTI) to calculate stroke volume (SV), then multiplies by heart rate (HR) |
| **Measurements** | Beat-to-beat, continuous | Typically, intermittent |
| **Parameters considered** | Age, gender, body mass, MAP, systemic vascular resistance | LVOT diameter, blood flow velocity |
| **Invasiveness** | Non-invasive | Non-invasive |
| **Calibration of equipment** | May require calibration with thermodilution for flow signal | Requires accurate measurement of LVOT diameter and assumes laminar flow |
| **Advantages** | Continuous monitoring, provides various hemodynamic parameters – validated to assess variations brought about by acute stress testing. | Widely available, relatively easy to perform (yet not performed in the current study) as limited availability in South Africa. |
| **Limitations** | Relies on assumptions, may be affected by additional factors, variable accuracy compared to thermodilution | Assumes laminar flow, accuracy depends on operator skills, can be affected by acoustic windows and patient factors |
| **Algorithm components** | Modelflow algorithm to estimate cardiac output (CO) from a peripheral arterial blood pressure (ABP) waveform. This algorithm computes the aortic flow waveform by simulating a non-linear three-element model of aortic input impedance  **Modelflow Details:** Modelflow is a development of the original corrected impedance (cZ) method. Assuming constant impedance (Z) based on age and mean arterial pressure (MAP), this approach uses the systolic segment of the pressure waveform to calculate stroke volume (SV) from the integral of the pulsatile area (PSA). It was subsequently updated with a 3-element Windkessel model. | Echocardiography calculates CO by measuring stroke volume (SV) and multiplying it by heart rate (HR). SV is determined by measuring the cross-sectional area of the left ventricular outflow tract (LVOT) and multiplying it by the velocity time integral (VTI) of blood flow through the LVOT.  CO = SV x HR  SV = LVOT area x LVOT VTI. |
| **Assumptions** | Derives 18 cardiovascular parameters, including SV, CO, and Systemic Vascular Resistance (SVR).  **Calibration:** Cardiac output derived from the flow signal may require a calibration with thermodilution. | This method assumes that blood flow through the LVOT is laminar and that the LVOT diameter is accurately measured |

**Based on information from the following sources:**

Waldron M, David Patterson S, Jeffries O. Inter-Day Reliability of Finapres ^®^ Cardiovascular Measurements During Rest and Exercise. Sports Med Int Open. 2017 Nov 17;2(1):E9-E15. doi: 10.1055/s-0043-122081. PMID: 30539112; PMCID: PMC6225956.

Sun JX, Reisner AT, Saeed M, Heldt T, Mark RG. The cardiac output from blood pressure algorithms trial. Crit Care Med. 2009 Jan;37(1):72-80. doi: 10.1097/CCM.0b013e3181930174. PMID: 19112280; PMCID: PMC3107992.

FinapPress Basic User Manual. FMS. Document ID: 0283-02-001-EN (910700) Version 1.9 Version date: 04 April 2016.
